# A Novel Advantage to Microtia Reconstruction Utilizing Ear Molding: A Case Report of Goldenhar Syndrome's Clinical Presentation and Surgical Reconstruction

**DOI:** 10.7759/cureus.81275

**Published:** 2025-03-27

**Authors:** Aneeq S Chaudhry, Yulia Shtanko, S. Anthony Wolfe, Sheryl Lewin, Martha Mejia

**Affiliations:** 1 Plastic and Reconstructive Surgery, Florida International University, Herbert Wertheim College of Medicine, Miami, USA; 2 Plastic and Reconstructive Surgery, Nicklaus Children's Hospital, Miami, USA; 3 Plastic and Reconstructive Surgery, Lewin Ear Reconstruction, Torrance, USA

**Keywords:** congenital, craniofacial microsomia, ear molding, goldenhar syndrome, microtia repair and auricular reconstruction, plastic and reconstructive surgery, surgical case report

## Abstract

This case report describes a unique approach to reconstructing microtia in a patient with microtia and craniofacial microsomia. Microtia is a congenital outer ear deformity that can vary in severity, from minor abnormalities to complete absence of the ear. The traditional methods for microtia reconstruction are autologous reconstruction using the patient's rib cartilage or alloplastic reconstruction using porous polyethylene. Autologous reconstruction typically begins around age six to 10 years of age when the rib cage is sufficiently large enough, while alloplastic reconstruction may be performed as young as three years of age.

In this case, the surgeons used ear molding in infancy to alter the shape of the existing skin and cartilage, thus creating a larger surface area for the ear. This approach, which is not commonly used, aims to create a more favorable shape for the microtia remnant to assist in the eventual reconstruction. A subsequent procedure to elevate the microtic ear was performed at the time of the surgical repair of macrostomia (lateral cleft lip).

Using ear molding in infancy for microtia reconstruction has several potential benefits. It can lead to a more natural ear appearance as the child ages, which can have positive psychological and social implications. By starting the reconstruction process early, the child may grow up with a less noticeable deformity. Finally, ear molding can also improve the shape of the newborn microtia ear leading to more favorable anatomy prior to surgery.

This case report highlights the advantage of early intervention with ear molding for selective patients with grade I or grade II microtia in preparation for their future reconstruction, particularly in patients with associated syndromes who may have more unique anatomy.

## Introduction

Microtia is a congenital malformation of the auricle that ranges in severity. The most widely used classification system, described by Marx and modified by Rogers, includes four grades [1]. Grade I describes a small but nearly normal ear, while grade II involves partial ear formation with some identifiable landmarks. Grade III is characterized by small auricular remnants, and grade IV, or anotia, refers to the complete absence of the external ear and ear canal.

Microtia can occur in isolation or in association with a syndrome. The most common syndrome seen with microtia is hemifacial microsomia (HFM), which affects the development of the lower half of the face, including the mandible, the mouth, the ear, and the soft tissues of the face. When the cranial bone is also affected, the term craniofacial microsomia (CFM) is used. Goldenhar syndrome (GS) is a variant of HFM that is associated with cardiac and renal anomalies, epibulbar dermoids, and vertebral malformations.

The incidence of microtia in the United States is 1.8 to 3.5 in every 10,000 births and 0.4 to 8.3 worldwide with certain ethnic groups showing higher rates [2]. Microtia is often associated with an abnormal ear canal and conductive hearing loss. Aural stenosis refers to a small canal with decreased hearing, while aural atresia is the complete absence of an ear canal.

Patients with microtia have several options for treatment, including autologous reconstruction with rib cartilage, alloplastic reconstruction with high-density porous polyethylene, or the use of a prosthetic ear.

Existing literature shows that the incidence of ear deformities in infants has been documented between 6% and 58% [3]. Deformations of the ear are instances in which the ear develops normally but is misshapen at birth. The maternal hormones circulating in the neonate allow for the malleability of the ear cartilage in the newborn. These neonates can be treated in the first weeks of life with an infant ear molding system to permanently reshape the ear cartilage into a normal configuration [4]. Infant ear molding can reduce the need for surgical intervention later in life and minimize the psychological distress associated with misshapen ears.

Neonates born with grade III or IV microtia cannot utilize infant ear molding as there is not any cartilage to reshape. However, grade I and grade II microtia represent a less severe form of this condition and may have some cartilage components that can benefit from ear molding.

## Case presentation

**Figure 1:**
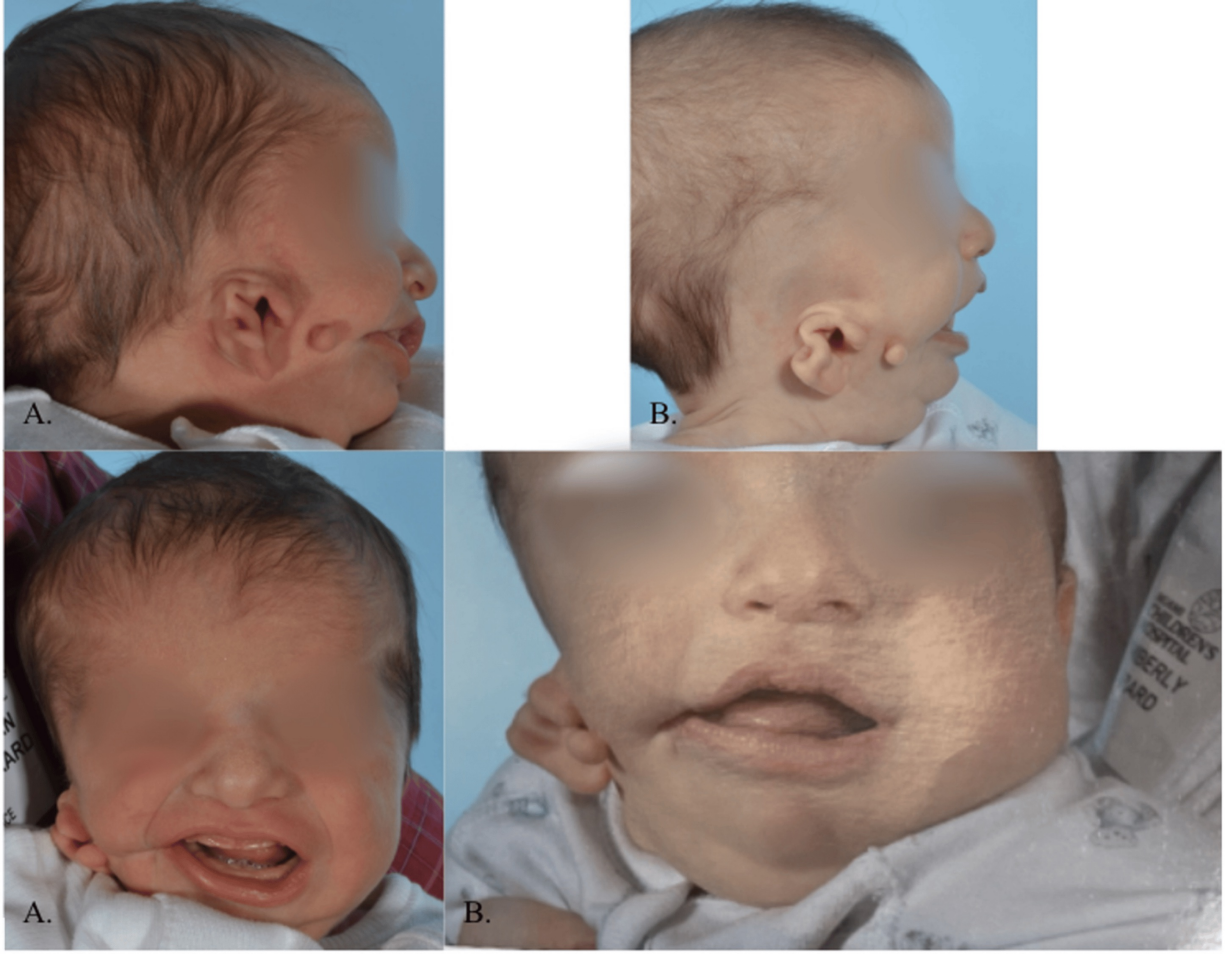
Pre- and post-molding progress. (A) Two weeks old (05/30/2012), pre-molding photograph. (B) Three months old (08/15/2012), post-molding photograph.

The patient was born at full-term via cesarean section with multiple abnormalities on the right side of his face, including grade II microtia, aural stenosis with conductive hearing loss, cranial microsomia, cheek chondrocutaneous remnant, macrostomia, and facial asymmetry (Figure 1A). He had no significant prenatal, intrapartum, or postnatal events, except for citalopram use during pregnancy. There was no family history of any similar condition.

At two weeks old, ear molding was applied to reshape the abnormal remnant and help pull the tissues upward over a three-month period (Figure 1B). This allowed the cartilage to be reshaped to provide an improved appearance until the patient was old enough to undergo surgical interventions.

**Figure 2:**
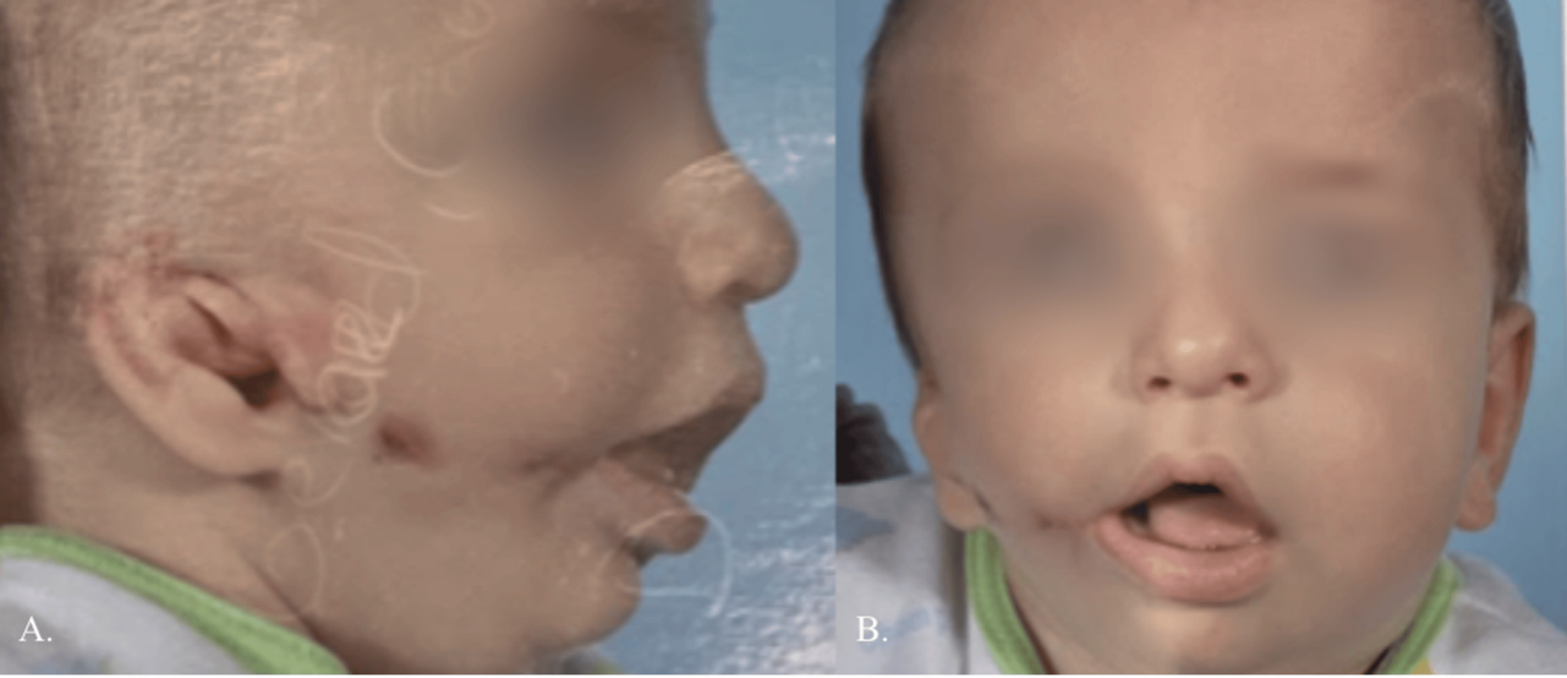
Five months old after ear molding, ear pexy, excision of chondrocutaneous remnant, and macrostomia. Photo taken on 10/10/2012.

At three months, the patient underwent macrostomia repair, excision of the preauricular chondrocutaneous remnant, upper and lower frenulectomy, and pexy of the right grade II microtia remnant (Figure 2).

**Figure 3:**
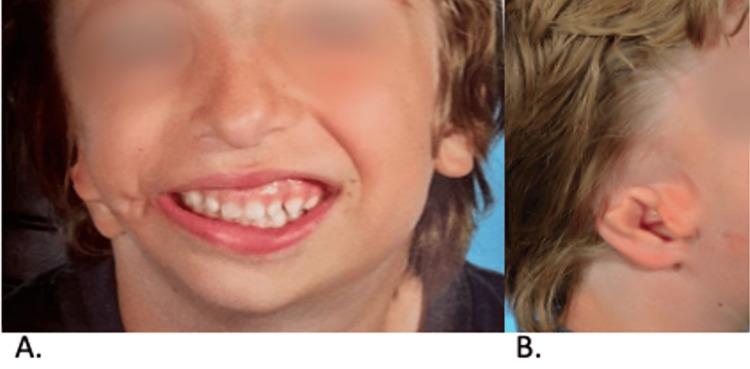
The patient at 4.5 years old. Approximately four years post-ear molding, ear pexy, excision of chondrocutaneous remnant, and macrostomia repair. Photo taken on 11/9/2016.

This procedure helped improve the positioning of the ear in preparation for future ear reconstruction while increasing the patient's self-esteem and quality of life throughout the next four years (Figure 3).

At five years old, he returned to surgery for revision of the tragus and macrostomia, as well as a fat injection to the right cheek to treat his hemifacial microsomia. Approximately 30 mL of autogenous adipose tissue was harvested from the medial thighs and abdomen. The adipose tissue was centrifuged and then injected through several small stab incisions into the right cheek, right preauricular area, right parasymphyseal area, and right temporal area.

**Figure 4:**
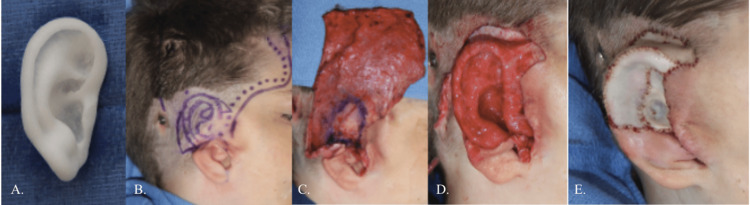
Porous implant ear reconstruction (PIER). A customized porous polyethylene implant was created from 3D imaging of the unaffected left ear (A). The implant was covered with a large temporoparietal fascial flap (B-D) and full-thickness skin grafts from the opposite ear and abdomen (E).

The patient went to another institution for porous implant ear reconstruction (PIER) at eight years of age. His grade II microtia and aural stenosis were significantly lower than the opposite ear, necessitating building the ear much higher than the canal could reach. The reconstruction was designed to improve facial symmetry, considering the patient’s craniofacial microsomia and low-set aural stenosis. Figure 4 illustrates the 1st stage of PIER. A customized porous polyethylene implant was created from 3D imaging of the unaffected left ear (Figure 4A). The implant was covered with a large temporoparietal fascial flap (Figures 4B-4D) and full-thickness skin grafts from the opposite ear and abdomen (Figure 4E).

**Figure 5:**
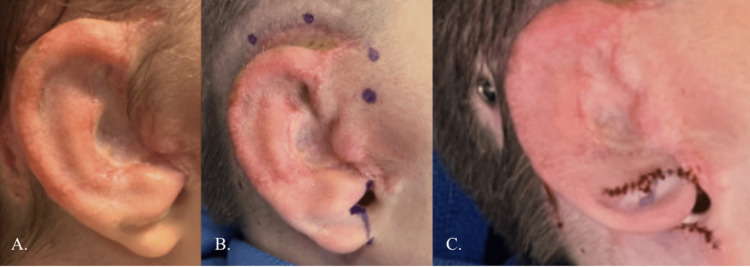
Stage 1 and stage 2 porous implant ear reconstruction (PIER). (A) Three-month postoperative appearance after stage 1 PIER. (B) Intraoperative plan with native canal and posteriorly malpositioned earlobe. (C) Immediate postoperative appearance after stage 2 PIER.

**Figure 6:**
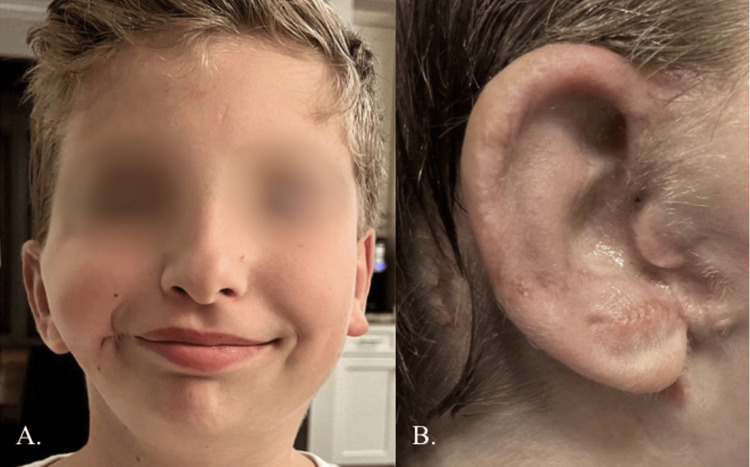
Progress after stage 2 porous implant ear reconstruction (PIER). (A) Three months after 2nd stage PIER. The PIER ear is still lower than the opposite side but is improved. (B) The new earlobe position hides the transposed native ear canal.

Three months after the 1st stage PIER, the patient underwent a second stage to elevate the ear superiorly, and a modified z-plasty to simultaneously transpose the native external auditory meatus posteriorly and the earlobe anteriorly to camouflage the abnormal position of the low canal (Figures 5, 6).

## Discussion

**Figure 7:**
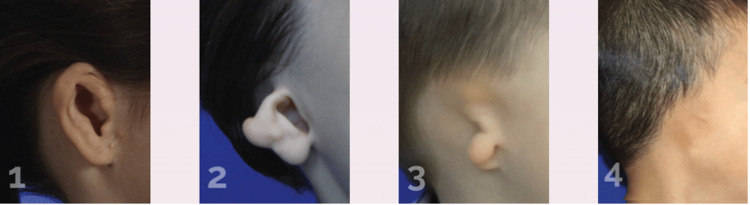
Grade I-IV Lewin ear reconstruction. 1. Grade I: Small but almost normal ear. 2. Grade II: Small ear with some recognizable anatomy, and the canal may be normal or small. 3. Grade III: Small remnant of soft tissue with no ear canal. 4. Grade IV: No external ear and no ear canal (anotia). Figure Credits: Sheryl Lewin. Lewin Ear Reconstruction [5].

Early intervention with ear molding can significantly enhance the quality of life and self-esteem of children with microtia, particularly in Goldenhar syndrome cases, which often present with complex facial anomalies. Microtia, characterized by the underdevelopment or absence of the external ear, is classified into four grades based on severity (Figure 7) [1,5]. Microtia can profoundly affect a child’s appearance and psychological well-being [6]. Despite the effectiveness of ear molding for correcting milder auricular deformities, there is limited research on its application for microtia [7]. However, studies have shown that early interventions with ear molding can improve the aesthetics and therapeutic value for mild microtia, providing a foundation for subsequent reconstructive plastic surgery procedures [8].

Ear molding involves using tapes, glues, stents, and custom-made shapes to gently stretch the ear's skin and cartilage. This technique takes advantage of the ear's malleability in infancy, which is attributed to decreased auricular muscle activity due to increased estrogen levels. Importantly, ear molding is a cost-effective and non-invasive option.

Early initiation of ear molding can mitigate the psychological impact of microtia, particularly in older children who may have already experienced social pressures and negative self-image [6,9]. By improving the ear's shape early on, ear molding can enhance self-esteem and reduce the need for more extensive surgical interventions later in life.

In the case presented, ear molding was initiated at three weeks of age, allowing for the gradual reshaping of the ear's cartilage and skin. This approach led to more natural and regular ear anatomy, which likely contributed to improved patient self-esteem and quality of life. Subsequent surgical interventions, such as PIER, successfully provided a cosmetically pleasing outcome.

## Conclusions

This case highlights the efficacy of early ear molding in improving the anatomical structure of the external ear in microtia, especially in Goldenhar syndrome cases. Early intervention with ear molding can have significant psychological benefits. This approach may reduce the need for more invasive surgical procedures later in life while offering a foundation for subsequent reconstructive treatments. These findings underscore the importance of integrating early, non-invasive techniques into the management of congenital ear anomalies to optimize long-term outcomes.
